# Unleashing their potential: a critical realist scoping review of the influence of dogs on physical activity for dog-owners and non-owners

**DOI:** 10.1186/1479-5868-8-46

**Published:** 2011-05-21

**Authors:** Ann M Toohey, Melanie J Rock

**Affiliations:** 1 Population Health Intervention Research Centre, University of Calgary, Teaching, Research and Wellness Building Room (3rd floor), 3280 Hospital Drive NW, Calgary, AB, T2N 4Z6, Canada; 2 Population Health Intervention Research Centre, University of Calgary, Teaching, Research and Wellness Building Room 3E15 (3rd floor), 3280 Hospital Drive NW, Calgary, AB, T2N 4Z6, Canada

## Abstract

**Background:**

Dog-owners tend to be more physically active than non-owners; however, dogs have also been shown to inhibit physical activity for non-owners, under some circumstances.

**Methods:**

We conducted a scoping review to identify studies pertaining to the influence of dogs on physical activity for both dog-owners and non-owners, and adopted a critical realist orientation to draw inferences about the positive and negative impact of dogs via their affect on physical and social environments.

**Results:**

We identified 35 studies from disparate literatures for review. These studies confirm that dog and owner behaviors affect shared physical and social environments in ways that may influence physical activity patterns, not only among dog-owners but also among non-owners. The direction of influence appears to be most positive in neighborhoods exhibiting high levels of social cohesion, socioeconomic status, perceived safety, dominant culture, or all of these. In disadvantaged neighborhoods, the health of women as well as older adults may be disproportionately affected by dog and owner behavior.

**Conclusions:**

While dogs have the potential to increase physical activity for both dog-owners and non-owners, the presence or absence of dogs will not have a standard effect across the physical and social environments of all neighborhoods. Dogs' contributions to shared environments in ways that support physical activity for all must be leveraged. Thus, specific contextual factors must be considered in relation to dogs when planning neighborhood-level interventions designed to support physical activity. We suggest this population health topic merits further investigation.

## Background

Amidst growing concerns over inadequate levels of physical activity that prevail in Western, industrialized countries - and the impact this trend is having on population health - a small but growing evidence-base has begun to explore dogs as facilitators of regular and sustained walking behaviors for their owners. Results have suggested that dog-owners tend to be more active than non-owners, in part because they regularly exercise their dogs [1-3]. At the same time, research into environmental correlates of physical activity has considered dogs to be potential barriers to physical activity for some (e.g., non-dog-owners [4]). These findings appear to oppose one another, suggesting that dogs may benefit some while putting others at a disadvantage in terms of achieving adequate levels of physical activity to support health. Because more than one-third of urban households in Western countries currently include dogs [5-8], their potential to influence population health for dog-owners as well as non-owners merits further investigation.

Interactions between non-owners and dogs tend to occur in public spaces, including neighborhood sidewalks, pathways and parks. A small handful of studies have extended the exploration of dogs as a motivation for owners to walk regularly by examining whether dog-owners' perceptions of the physical and social environments of their neighborhoods differs from non-dog-owners' perceptions [5,9]. However, the evidence suggesting dogs play a role in how people perceive their neighborhoods is not limited to studies directly exploring dog-owners. We have noticed occasional, unsolicited mention of dogs - whether their own, or someone else's - by participants in qualitative research studies that consider relationships between neighborhoods and health, stemming from both physical activity and social capital literatures. Because neighborhood perceptions, including safety [4,10] and social cohesiveness [11], have been linked with physical activity, we began to suspect that the influence of dogs on physical activity may well extend beyond just the owner, and could in fact have an effect upon non-owners who share and negotiate physical and social environments with dogs and their owners.

Our objective, in conducting this review, is to pull together evidence found in diverse literatures to deepen our understanding of dogs' potential to influence physical activity for both dog-owners and non-owners through dogs' impacts on shared social and physical environments. We report on a scoping review [12,13] informed by critical social theory. Our definition of 'society', which typically is limited to human populations, is extended to include non-human animal populations [14,15], and given our interest in urban environments, we are particularly concerned with pet dogs as actors within social contexts. We extend our definition of society to include dogs because more than 1 in 3 urban households in Western and some non-Western countries include dogs, and most of these dog-owners regard their dogs as family members [16,17]. In keeping with a critical realist approach [18], we view social structure as having the potential to propagate health inequalities through unequal distribution of material and symbolic resources, yet we acknowledge that individuals' experiences within these structures may be varied and influenced by agential, or intrapersonal, factors. To synthesize the findings from the studies included in our scoping review, we adopted the realist review methodology championed by Pawson and colleagues [19], which considers "What works, for whom, and in what circumstances?" This approach enabled us to identify a range of insightful studies, to explore both the structural and agential aspects of study results, and to advance propositions regarding what sorts of interventions might be most effective and most needed in different social contexts.

## Methods

### Inclusion criteria

We included primary research pertaining to the influence of dogs on physical and social environments in ways that plausibly impact physical activity behaviors for both dog-owners and non-owners, such as neighborhood-based walking. Studies were not excluded based on their methods, nor on any assessment of their methodological rigour. The scope was limited to studies written up in English, conducted in industrialized Western countries, and published in peer-reviewed journals between 2000 and 2010. Studies of the impact of dogs on the health of individual owners, with little or no discussion of the broader implications of their potential impact on factors contributing to physical and social environments, were excluded; these are discussed elsewhere [16,17,20-22]. Review articles were excluded but their reference lists were used to identify pertinent articles. Titles, abstracts and results were screened for inclusion by the lead author, in consultation with the other author.

### Search strategy

We searched PubMed for references containing one or more of the following combinations of words in the title, abstract or keyword fields: i) neighborhood *and *social capital; ii) neighborhood *and *walkability; iii) neighborhood *and *dogs, iv) walkability *and *intervention, v) social network analysis *and *veterinary. Anthrozoology.org was used to find relevant publications within 'animals & social support' and 'animals & elderly people' topic areas. We later searched PubMed, as well as PsycInfo, LeisureTourism Abstracts and Web of Science, to find qualitative studies that included the following keywords or phrases: (physical activity, exercise, inactivity, walking, *or *motor activity) *and *(environment, neighborhood, urban design, park, trail, greenway, *or *environmental design). We limited this follow-up search to qualitative studies because a preliminary analysis identified qualitative studies in which dogs figured in the results in insightful ways, but dogs were not highlighted in the introduction, discussion, abstract, title or keywords.

We then used Web of Science to conduct citation searches on several fundamental articles [4-6,9,21,22]. To help ensure that no relevant papers were overlooked, hand-searches were performed on two journals specializing in the study of human-animal interactions (namely, *Anthrozoös *and *Society & Animals*) for articles that included 'social' or 'neighborhood' in their titles. Finally, we also looked for articles by consulting reference lists of the studies meeting the inclusion criteria and relevant review articles, as well as through contacts with information science, physical activity, social capital and anthrozoology experts, and in the course of other research projects.

### Data extraction

As informed by our critical social science orientation, the data contained within the articles included in our review describes the human experience with dogs within contained physical and social environments [14,15,18]. All articles were reviewed and summarized using a standard data extraction form designed to capture this discursive evidence, based on what study participants reported or expressed (see Additional File 1). We found it helpful to use the realist review strategy of "What works, for whom, and in what circumstances" [19] to guide our data extraction and synthesis. Both authors read each study independently. The lead author extracted the following information: the main purpose of the study; the study population; notes of interest on the physical and social context of the study; theoretical framework; whether a formal intervention was being evaluated; methodology; results; implications for social capital; implications for physical activity; and whether possibility of dog-related conflict was addressed. This author also constructed a table identifying the study type, demographics, the intervention being evaluated, results of relevance to the present study, and implications regarding physical activity for both dog-owners and non-owners. The other author independently verified data extraction and tabulation for the included articles. Both authors met regularly over a period of approximately 24 months to discuss findings. In instances of disagreement, articles were reassessed independently and consensus was reached following deliberation.

### Analysis

As outlined by Willig's helpful overview of critical realist epistemology and ontology [18], we analyzed the human experience extracted from our population of studies in terms of physical activity, which we consider to be a category from medicine and public health discourses. Central to critical realism is the notion that "what appears to be the 'same' unit may display very different characteristics in different contexts" [18], a tenet that is also central to recent contributions to actor-network scholarship on science, medicine, geography and animal-human studies [23-27]. Thus, foundational to our analytic strategy was attention to the ways that dogs were viewed differently, or played different roles, in different contexts. We paid attention, more specifically, to relationships between dogs and dog-owners; among dog-owners; between dogs and non-owners; and between dog-owners and non-owners. For example, we analyzed neighborhood-based walking as a practice [18] in which dogs often participate, with implications for physical activity for both dog-owners and non-owners.

Throughout the review process, we speculated on how this information might inform alternative futures [18], through interventions designed to improve health but also interventions such as urban planning and management that are not usually designed chiefly with health in mind [28]. This analytic approach involved constant comparison across included and candidate studies, to distill key themes [13]. As illustrated by Table 1, this line of inquiry was consistent with our focus on contextual factors, vis-a-vis social and physical environments, that can influence population health and the impact of interventions, whether designed by health researchers or resulting from change processes outside of the health sector [28].

**Table 1:** Framework for applying a realist orientation to reviewing evidence in population health intervention research

***Orientation of Population Health Intervention Research (adapted from Hawe & Potvin, 2009*) **[28]		***Orientation of Realist Approach (adapted from Pawson et al., 2005) ***[19]
PLAN	Theorize about change dynamics and draw causal inferences. Assess all interventions and factors in place that merit consideration as contributors to preventing incidence.	Paying particular attention to contextual factors as they relate to expected health outcomes for different populations, use empirical evidence from the literature as well as practice-based wisdom to inform planning processes.

IMPLEMENT	Be sensitive to stakeholder and end-user needs, which may differ significantly from researchers' goals. While interventions must be replicable, they may require revisions to be effective in different contexts. Practice the 'duty of care' for all participants in the intervention.	Gain insight into contextual factors and particularly mechanisms by which interventions may work differently in different contexts. Consider which potential outcomes might be expected for different populations and sub-populations, and strategize means of tailoring implementations to be effective for different populations.

EVALUATE	Evaluate the intervention for: - Relevance; - Coherence between theory of problem and theory of change; - Responsiveness to local conditions; - Achievements, particularly health outcomes; - Results and impact, or the relationship between the intervention and the change.	Use both supporting and contradicting evidence to gain further insight into the effects of context upon efficacy and impact of interventions for different populations and sub-populations.

## Results

The PubMed searches yielded 1,002 references for consideration, and of these, **4 **were included. Hand-searches of *Anthrozoös *yielded 9 references, **3 **of which were included, and *Society & Animals *yielded 12 references, **1 **of which was included. From the physical activity literature, we added **8 **articles. Related research projects, professional contacts and citation searches yielded an additional **19 **articles. In all, we included **35 **articles for review.

These 35 studies represent a heterogeneous sample of populations and methodologies. As an ensemble, they offer rich information on dogs' potential to influence physical and social environments in ways that impact physical activity behavior for both dog-owners and non-owners. Additional File 2:Table S1 lists the 35 included studies, outlines key results and distills implications for the health of human populations in relation to physical activity.

### Dogs and physical urban environments

Throughout the studies we reviewed, dogs were conceived as having impact on physical urban environments in two specific ways that pertain to physical activity: as a source of nuisance *via *dog litter in public spaces such as parks or sidewalks [6,29-33], and via stray or uncontrolled dogs [4,6,33-40]. Both of these were viewed as negative elements in the physical urban environment, generally correlated with lower levels of physical activity, for both dog-owners and non-owners. Both were also seen as affronts to safety.

For example, in evaluating a physical activity intervention targeting physically inactive adults, Sallis and colleagues found that women who reported loose or unattended dogs in their neighborhoods also reported approximately 50 minutes per week less physical activity compared to women who did not report this presence [4]. Participants in two separate focus group studies suggested enforcement of policies concerning loose or stray dogs might help increase physical activity levels in their neighborhoods [34,35]. Additionally, in ranking nuisances that led to decreased use of local parks, older adults participating in a study conducted by Alves and colleagues ranked dog litter as second only to vandalism in importance [31].

One of the studies in our review took a novel approach to comparing associations between perceived and objective environmental factors with two different physical activity outcomes: meeting recommended levels of physical activity (150 minutes/week), and walking for recreation [41]. These researchers included a collective measure of dogs living within a 0.8 km radius of respondents as an environmental factor, based upon dog licensing data. Results suggested that study participants living in areas with larger canine populations were also more likely to walk. (This association held only when comparing the lowest and the middle tertile of dogs; the highest tertile did not reach significance.) As the sample was drawn randomly, this finding pertained to both dog-owners and non-owners, but the authors did not compare physical activity behaviors for dog-owners and non-owners.

### Dogs and social urban environments

#### i) Dogs as motivators of physical activity for dog-owners and non-owners

By extending our definition of family and society to acknowledge the presence and importance of dogs, we were able to view dogs as contributing a social dimension to peoples' lives. As such, social norms surrounding responsible dog-ownership were often associated with dog-walking behaviors, and the dogs themselves were directly attributed with increasing the amount of walking for their owners in several studies [1,2,5-7,9,42-49]. Participants in Knight & Edwards' study of older adult dog-owners discussed how their dog motivated them to overcome such barriers as minor illness or depression, lethargy, bereavement, insecurity about walking alone, and inclement weather to ensure that the dog was exercised [7]. Furthermore, they reported inevitably 'feeling better' for having done so. In a study of the mobility of older adults, Thorpe and colleagues compared dog-owners and non-owners who walked comparable amounts at baseline, and found that the dog-owners were twice as likely to continue to achieve 150 minutes/week of walking three years later [1]. Cutt and colleagues measured changes in weekly walking behaviors for adults who acquired dogs over a year from baseline, compared to those who did not. They found that new-dog-owners increased their weekly walking for recreation by 36 minutes more than those who had not acquired a dog, and increased their total weekly walking by 33 minutes more than those who had not acquired a dog [42]. Adult participants in Wood et al.'s study exploring associations between pets and social capital in Western Australian suburbs also reported that owning a dog encouraged them to walk more within their neighborhood than they would otherwise do [9].

We were surprised to also find a small number of studies where dogs were a source of sustained physical activity for non-owners as well [43,50]. Johnson & Meadows designed an innovative dog-walking program to explore whether adherence to a walking intervention could be improved by involving 'loaner' dogs. The walking program targeted two separate groups of public housing residents who were able to walk independently, and who were not afraid of dogs. The program also enlisted certified therapy dogs and their handlers. Overall adherence was high: of the original group of 30 participants, only 8 withdrew, but 4 of these reported schedule conflicts, health issues, or re-location as reasons for leaving the program. All remaining participants achieved walking levels of 20 minutes/day, 5 days per week. It became clear that they were motivated by the commitment they felt to the dogs, and their belief that the dogs needed them. Furthermore, several of the participants' comments suggested that - similar to Knight & Edwards' findings [7] - walking the dogs also made them feel better [50].

Peel and colleagues' study of people with type 2 diabetes, which focused on physical activity in their daily lives, revealed unexpected findings regarding the influence of dogs in social environments [43]. Dogs were viewed as assisting with the maintenance of a physical activity regime; meanwhile, other efforts to become more active attenuated with time. One woman, with an admitted lack of interest in physical activity, tried several different programs over the course of the study. None were successful, until - by the 4^th ^year - she began to join a neighbor's regular dog-walks. While this individual did not own the dog, she clearly benefitted from the regular dog-walking practices of her neighbor, which became a source of motivation and routine for her.

#### ii) Catalysts of social interactions leading to increased social cohesion

Neighborhood social cohesion has been positively associated with levels of physical activity [11], and having positive social interactions within neighborhoods helps build social cohesion. Several of the studies in our review provided evidence that dogs facilitate increased social interactions in a variety public spaces, including parks, sidewalks, and pathways, as well as public transit facilities, school drop-off areas, and outdoor malls [5-7,9,33,44,45,51-53]. The controlled experimental studies that measured quantity and quality of social interactions resulting from the presence of a dog confirmed that these were initiated by the 'other' as opposed to the dog-owner or the dog itself [51,52]. Dog-owners themselves were aware of this role that dogs played, but in a focus group study by Wood and colleagues, a non-owner also acknowledged the social opportunities that arose from seeing regular dog-walkers in his neighborhood [5]. Participants in Knight & Edwards' focus group study, who were dog-owners themselves, suggested that without a dog, there would be no informal interactions with strangers in their neighborhood [7].

#### iii) Relationships with neighbors

Many of the studies we reviewed suggested that dogs often played a positive role in establishing and strengthening relationships with neighbors [5,6,9,33,43,46,54], which can also contribute to positive perceptions of neighborhood social cohesion. Reciprocity in terms of pet care was one mechanism by which these relationships evolved [54]. In fact, Wood and colleagues noted that mutual aid between dog-owners and neighbors involved favours that did not directly involve dogs or any other pets [9].

Yet we cannot assume that all dog-related influences on neighbor-relations will be positive in nature: Bjerke & Ostdahl's study found that nearly one-quarter of a random sample of participants reported 'problems with neighbors' dogs' [47]. Furthermore, a participant in Martinez and colleagues' study of Latina women mentioned being chased by her neighbor's unleashed dogs as a barrier to taking her children out and about in her neighborhood [36]. Dog-owners in Cutt and colleagues' study also identified uncertainty of neighbors' perceptions of dogs as a potential barrier to walking in their neighborhood [6].

#### iv) Companions on an informal neighborhood watch leading to increased sense of safety

Another relevant theme that arose in several studies was the role dog-walkers play in establishing a sense safety in the neighborhood through their regular presence and, as a result, heightened awareness of any unusual occurrences or dangers [5-7,9,46]. This has implications for neighborhood-based physical activity for all residents, dog-owners and non-owners alike, given established associations between the safety of the neighborhood and the likelihood of being physically active [10,38,55]. Such a role is illustrated in Boneham & Sixsmith's study of a community of older women living in a disadvantaged neighborhood [46]. A participant related how a neighbor's evening dog-walk revealed that another elderly neighbor had collapsed and was in need of immediate medical assistance. Using the daily task of walking a dog allowed residents to check on their neighbor's welfare, but without invading her privacy.

## Discussion

### Synthesis of findings

Most of the studies we reviewed either explicitly or implicitly adopted a social-ecological framework, recognizing the significance of interactions between intrapersonal, social, physical and cultural environments for perceptions of physical and social urban environments and, ultimately, physical activity behaviors [56,57]. By highlighting whether the influences of dogs on physical and social environments were positive or negative, who was impacted by these influences, and what characterized the physical and social contexts in which this was taking place, our attention was directed to assessing the overall impact dogs had on the sharing of public spaces. In particular, we considered processes that could render dogs into influencers of physical activity among dog-owners and non-owners of different ages and in different life stages.

The direction of influence was not uniformly or consistently positive. Women [4,34-39], ethnic minorities [34-40], and older adults [29,31,39] seemed to be most susceptible to experiencing other people's dogs as barriers to being physically active, although there were exceptions [45,50]. In contrast, studies focusing upon middle-class, predominantly Caucasian adult populations positioned other peoples' dogs as either having no impact on the physical activity patterns of women [58] or older adults [59], or as facilitators of physical activity through the positive contributions made to the social environment [5-7,9,33,41,54].

Notably, in King et al.'s intervention research on physical activity, loose and unattended dogs were identified as a barrier in ethnic-minority neighborhoods in Atlanta, Georgia and Memphis, Tennessee, while participants from Stanford, California; Eugene, Oregon; and Kingston, Rhode Island - primarily White populations - did not identify dogs as barriers [40]. This difference was not explained by household income levels, as the Oregon and Rhode Island samples had lower average household incomes than did the Georgia and Tennessee samples. Sallis and colleagues noted similar findings, stating the lowered levels of physical activity reported by women in neighborhoods where loose and unattended dogs were more likely to be reported were not explained by variations in socioeconomic status [4]. This suggests that social disadvantages associated with gender, age, and race/ethnicity may be more pertinent than income when predicting the direction of influence dogs might have on surrounding social and physical environments, which has implications for future interventions and evaluation of interventions.

Clearly, urban-dwelling dogs have the potential to impact the health of human populations in both positive and negative directions. The negative aspects of negotiating the use of shared spaces with dogs are modifiable, however, and could be leveraged in ways that can overcome not just the direct, dog-related outcomes, but other environmental factors that were often reported alongside nuisance from and fear of dogs. For example, many African American women who participated in Griffin et al.'s study [39] listed fear of uncontrolled dogs among the factors inhibiting them from being more active, but also proposed that increasing a sense of connectedness among neighbors was a potential solution to increasing physical activity. A campaign targeting responsible dog-ownership could potentially minimize the role dogs play as barriers and reposition them as facilitators of population-level physical activity. Similarly, in Sanderson and colleagues' study [35], African American adults identified seeing no one else out being active in the neighborhood as a barrier to being active themselves. These participants also suggested that living in 'a good neighborhood,' where neighbors looked out for one another, would facilitate exercising outdoors. An increased presence of responsible dog-owners - who walked their dogs regularly, kept them on-leash and under control, and picked up dog litter - in these neighborhoods could address both of these conditions, as was shown in studies by Wood and colleagues [5,9].

Lacking the motivation to be physically active was also identified by some study participants as contributing to physical inactivity [35,43]. And yet, dog-owners were able to overcome their own personal barriers, including minor illness and depression as well as inclement weather, to walk their dogs on a daily basis [7]. While we acknowledge that reasons for choosing not to own a dog may range from financial limitations, housing situations, and time-constraints to personal preferences, some of these could be addressed by physical activity interventions that leverage, to the benefit of non-dog-owners, the motivation dogs seem to provide to walk regularly. Peel and colleague's account of a type 2 diabetic who was able to access her neighbor's dog-walking regime to incorporate regular physical activity into her own life supports the supposition that ownership is not a requirement for someone to benefit directly from a dog's need to be walked. Many of the studies in this review offered evidence that dog-owners are more likely than non-owners to attain and to maintain recommended levels of physical activity, yet interventions could help increase the likelihood of non-owners achieving this important public health goal. In this regard, the results of Johnson & Meadow's 'loaner dogs' intervention among public housing tower residents are encouraging.

### Strengths and limitations of this review

Realist reviews have, to date, been used to evaluate the effectiveness of planned interventions [60,61] and, as far as we know, ours is the first to apply this method to a scoping review. Given the disparate literatures involved, as well as the observation that dog-relevant evidence is at times included in results, but not highlighted or discussed by the authors, it is possible that we did not identify all studies that met our inclusion criteria. Nevertheless, we did not exclude any study based on methodological grounds, as our aim was to be as inclusive as possible. This bias toward inclusion was helpful, given that the studies appeared in journals with divergent audiences and disciplinary orientations. Still, the total number of studies meeting our inclusion criteria remained manageable. It is also to be expected that previous experience in research and practice influenced our inclusion decisions and interpretations of the data, and so other researchers could adopt our search strategy and yet end up with different inclusion decisions and analytic interpretations [62].

### Implications for research and practice

Our analysis supports the need for a dedicated literature exploring the influence of dogs on population health via physical and social urban environments. Dogs, as our analysis highlights, do not have uniform effects and we need to understand more about these differences, so as to maximize benefits and minimize inconveniences and harms. In conducting this review, we wish to invite further intervention-oriented research on dogs as potential contributors to population health via physical activity.

When planning population health interventions that depend upon the social and physical qualities of neighborhood environments to achieve success, we strongly suggest looking beyond human populations. Responsible dog-ownership practices, as well as provision of dog-supportive amenities, are the key to enabling dogs to reach their potential as promoters of population health through their positive impacts on physical and social environments. Bjerke & Ostdahl's identification of the prevalence of problems with neighbors dogs, using a random sample rather than targeting disadvantaged communities [47], underscores the importance of designing, implementing and enforcing policy to reduce the negative and increase the positive benefits of co-existing with dogs. The findings of both Christian (neé Cutt) et al. [2] and Lee et al. [33] emphasized the importance of accessible, dog-supportive parks as arenas for becoming aware of and putting into practice responsible dog-ownership behaviors, in addition to encouraging walking (for both dogs and their owners) and facilitating social encounters with neighbors and other park-users. The presence of wildlife can encourage people to get outdoors and walk, which adds a layer of complexity in managing dog populations in urban environments and in large park settings [63].

It will be crucial, however, to consider strategies for ensuring that disadvantaged communities are not excluded from these efforts. These neighborhoods will likely require the greatest investment of time and other resources. Animal restraint ordinances, the most commonly suggested means of eliminating stray or uncontrolled dogs as barriers to physical activity, may be of benefit but should be stringently evaluated. Options that could be implemented and evaluated include: spay/neuter programs, subsidized veterinary services, pet food donations, responsible dog-ownership campaigns and training classes, dog-sharing and dog-fostering programs, amenities that support dog-walking, and enforcement of ordinances regarding dog litter and dog control.

## Conclusion

Dogs in our society have the potential to impact population health in both positive and negative directions. The presence or absence of dogs will not have uniform effects across physical and social environments: rather, contextual factors including physical, social, cultural, and political environments are key to predicting and shaping how dog populations will potentially impact physical activity for both dog-owners and non-owners. Similarly, identifying local policies for animal control enforcement is a circumstance that must be included in formulating such predictions. Future research should continue to study ways to maximize the positive and minimize the negative impact of dogs on population health in urban, suburban and peri-urban settings. Despite the complexity of the factors at play, reducing potential conflict and leveraging potential benefits of coexisting with neighborhood dogs will serve our population well as we strive to increase physical activity levels throughout the life course.

## Competing interests

The authors declare that they have no competing interests.

## Authors' contributions

AT and MR conceived of the study together. AT conducted the literature searches in consultation with MR. MR identified key papers and suggested search strategies based on her involvement in related research projects. Both authors read all papers under consideration and, over a course of 14 months, discussed which papers met inclusion criteria and the implications of the findings. AT constructed the initial draft of the manuscript and tabulated the data, and both authors worked together to revise and refine the synthesis until a final version was reached. Both authors read and approved the final version of the manuscript.

## Authors' information

MR is the director of the Population Health Intervention Research Centre at the University of Calgary: Her primary faculty appointment is with the Department of Community Health Sciences at the University of Calgary, and she holds a joint appointment in the Department of Ecosystem and Public Health in the Faculty of Veterinary Medicine. AT is a MSc student in Population and Public Health in the University of Calgary's Community Health Sciences department.

## Supplementary Material

Additional File 1 **Data Extraction Form**. The standard form which the authors used to extract relevant data from all studies included in this scoping review. Click here for file

Additional File 2 **Table S1 - Characteristics of Included Studies, Key Findings, and Implications for the Present Review**. A summary of studies included in this scoping review, tabulating for each the complete citation, methods, population and contextual factors, intervention being evaluated, key findings relevant to the present review, and implications regarding dogs' potential influence upon physical activity for dog owners and non-owners. Click here for file
