# Supplementary material for: Unleashing their potential: a critical realist scoping review of the influence of dogs on physical activity for dog-owners and non-owners
Source: Int J Behav Nutr Phys Act. 2011 May 21;8:46. doi: 10.1186/1479-5868-8-46 (PMC3123259; doi:10.1186/1479-5868-8-46)
Supplement: Additional File 1 — Data Extraction Form. The standard form which the authors used to extract relevant data from all studies included in this scoping review. [file 1479-5868-8-46-S1.PDF]

Additional File 1 – Data Extraction Form

REFERENCE - Citation information

1. What is the main purpose of the paper?

2. What is the study population?

3. Notes on context considerations.

4. Theoretical frameworks used or discussed

5. Intervention being evaluated

6. Methodology

7. Results

8. Applications re: social capital

    i. Diffusion of knowledge/information

    ii. Behavioral norms/reciprocity

    iii. Access to amenities, services

    iv. Psychosocial processes (mutual respect, networks, etc.)

9. Applications re: physical activity

10. Does this paper address possibility of conflict/tension?

11. Other comments
