# Supplementary material for: Unleashing their potential: a critical realist scoping review of the influence of dogs on physical activity for dog-owners and non-owners
Source: Int J Behav Nutr Phys Act. 2011 May 21;8:46. doi: 10.1186/1479-5868-8-46 (PMC3123259; doi:10.1186/1479-5868-8-46)
Supplement: Additional File 2 — Table S1 - Characteristics of Included Studies, Key Findings, and Implications for the Present Review. A summary of studies included in this scoping review, tabulating for each the complete citation, methods, population and contextual factors, intervention being evaluated, key findings relevant to the present review, and implications regarding dogs' potential influence upon physical activity for dog owners and non-owners. [file 1479-5868-8-46-S2.PDF]

Additional File 2: Table S1 - Characteristics of Included Studies, Key Findings, and Implications for the Present Review

Reference	Methods	Population and contextual factors, as reported by authors	Intervention (formal or informal)	Key findings of relevance to present review	Implications re: dogs' potential influence upon physical activity of dog-owners and/or non-owners
Alves S, Aspinall PA, Thompson CW, Sugiyama T, Brice R, Vickers A. Preferences of older people for environmental attributes of local parks: The use of choice-based conjoint analysis. Facilities. 2008;26(11/12):433-453.	Quantitative: mail-out cross-sectional survey; random sample using choice-based conjoint questions on preferences in an experimental design	Older adults (60 – 97 yrs) – N=237<br>Diverse population from urban, suburban, rural locales<br>20 local authorities from across Britain (17 from England; 2 from Scotland, 1 from Wales)	Physical urban environment - I'DGO (Inclusive Design for Getting Outdoors – a study of how urban planning and design impact on senior health)	Nuisance, including presence of dog litter, was identified as the most important barrier to local park use as well as use of walking routes to access parks.<br><br>Among possible nuisances, dog litter was ranked second in importance, after vandalism.	Dog litter as a form of nuisance provides a barrier to non-dog-owners using parks and walking routes for being physically active.<br><br>Dog litter may also prevent dog-owners who clean up after their dogs from using amenities where dog litter is perceived as excessive.<br><br>This emphasizes the importance of interventions and policy designed to reduce dog-litter in public areas.
Bjerke T, Ostdahl T. Animal-related attitudes and activities in an urban population. Anthrozoos. 2004;17(2):109-129.	Quantitative: mail-out cross-sectional survey; random sample on animal-related activities and attitudes/preferences	Adult residents – N=720<br>Inner city and suburban representation<br>Trondheim, Norway	Physical urban environment – neighborhood mix of human and wild and domestic animal populations	Walking with a dog was rated as the most important motive for walking outdoors among dog-owners.<br><br>Problems with neighbors' dogs were reported by nearly one quarter of respondents. While specific problems were not described, reference was made to UK National Statistics (2001), which reported that dogs/dog litter were perceived as an environmental problem by 1/3 of the population.	Problems with neighbors' dogs, including dog litter, could potentially impede physical activity by disrupting neighborhood-based social cohesion, which has a direct correlation with physical activity.
Boneham MA, Sixsmith JA. The voices of older women in a disadvantaged community: issues of health and social capital. Soc Sci Med. 2006;62(2):269-279.	Qualitative: semi-structured interviews and focus groups	Older women (55 – 82 yrs) – N=19<br>Socially disadvantaged neighborhood<br>Diverse representation based on mobility, health and marital status, socially isolated and socially active<br>Northern town in England<br>(Specific location was not disclosed by authors)	Social urban environment – neighborhood composition	Dogs were identified as a motivation for dog-owners to walk in their neighborhood.<br><br>Dogs facilitated reciprocal favours between neighbors (taking care of each other's pets)<br><br>Dogs enabled residents to practice patterns of watchfulness – walking the dog enabled vigilance without invading privacy.	Reciprocity among neighbors and watchfulness both contribute positively to the social environment by contributing to both social cohesion and sense of safety in ways that can support increased physical activity for both dog-owners and non-owners within the neighborhood.

Chad KE, Reeder BA, Harrison EL, Ashworth NL, Sheppard SM, Schultz SL, et al. Profile of physical activity levels in community-dwelling older adults. Med Sci Sport Exer. 2005;37(10):1774 - 1784.	Quantitative: cross-sectional survey, convenience sample recruited from retirement complexes, older adult housing units, and community groups, self-administered	Older adults (50+ yrs), 95% Caucasion – N=764<br>Community-dwelling in a midsized Canadian city<br>(Specific location was not disclosed by researchers)	Physical urban environment - factors moderating physical activity patterns	Walking was a major contributor to the physical activity scores of respondents, although no data was collected as to whether respondents owned or walked dogs.<br><br>Most respondents did not report seeing unattended dogs in their neighborhoods, but the more active respondents were more aware of loose/stray dogs, i.e., a positive association was measured. (Also see King et al.'s (2000) results.)	While in other studies, awareness of loose/stray dogs resulted in a decreased sense of neighborhood safety, for this particular sample, reports of loose/stray dogs in the study neighborhoods were infrequent.<br><br>This is the type of neighborhood environment where we might expect dogs to have a potentially positive impact on social cohesion and sense of safety, thus supporting rather than inhibiting physical activity for all residents.
Christian nee Cutt, H., Giles-Corti, B., & Knuiman, M. (2010). "I'm Just a'-Walking the Dog" correlates of regular dog walking. Fam Community Health, 33(1), 44-52.	Quantitative: self-administered questionnaires at baseline and 12 months including a survey tool that was completed by all dog-owners (those who did not walk their dogs are excluded from this analysis)	Adult dog-walkers (who reported owning a dog and spending some amount of time walking with the dog)<br>N=483<br>Suburban residents of new housing estates Perth, Western Australia	Physical urban environment - dog walking among residents of new housing estates designed to promote more walking, cycling and use of public transit	Regular dog-walkers (participating in a minimum 90 min/week walking or jogging in the neighborhood with their dog) were more likely to walk dogs to local destinations.<br><br>Two-thirds of regular dog-walkers met recommended levels of physical activity (150 min/week) compared to just over a quarter of irregular dog-walkers.<br><br>Dog-owners living near dog-supportive parks (equipped with dog-related signage and litter bags for picking up dog litter) were more likely to be regular dog-walkers.	Dog-walkers' visits to local destinations could result in a regular presence on neighborhood streets, which could have a positive impact on the social environment of the neighborhood through social interactions and familiarity among neighbors.<br><br>Provision of local dog-friendly destinations might play a role in minimizing dog-related nuisances. Displaying dedicated signage could ameliorate encounters with uncontrolled dogs, and providing supplies and bins for convenient removal of dog litter could ensure that this is done. These are two dog-related factors that otherwise risk impeding physical activity for both other dog-owners and non-dog-owners.

Cutt HE, Giles-Corti B, Wood LJ, Knuiman MW, Burke V. Barriers and motivators for owners walking their dog: results from qualitative research. Health Promot J Austr. 2008;19(2):118-124.	Qualitative: 7 focus groups with dog-owners who had completed RESIDE baseline questionnaire	Adult dog-owners (23-73 yrs) – N=51 Suburban residents of new housing estates Perth, Western Australia	Physical urban environment - RESIDE Project (RESIDential Environments – a prospective study of physical activity levels among residents of new housing estates designed to promote more walking, cycling and use of public transit)	Dog-owners were motivated by social norms surrounding responsibilities of caring for a pet to walk their dog, pick up after their dog, and leash their dog when necessary.<br><br>Seeing others out walking, and the dogs themselves, were both identified as motivators for dog-owners to walk their dogs.<br><br>Barriers of dog-walking included fears that the dog-owner or their pet dog might be attacked by loose or uncontrolled dogs ; uncertainty of neighbors' perceptions of dogs; and lack of adherence by others to pet-related behavioral norms, and particularly cleaning up dog litter while out walking.<br><br>Benefits of dog-walking included improved sense of both collective and individual safety; meeting people, and getting to know neighbors and neighborhoods.	Dog-owners were motivated by seeing others out walking; seeing others out walking has also been identified as a motivation for physical activity by non-dog-owners.<br><br>Improved sense of both collective and individual safety; meeting people, and getting to know neighbors and neighborhoods were identified as benefits of dog-walking. These have all been correlated positively with neighborhood-based physical activity, regardless of dog-ownership status.<br><br>Dog behaviors that arise from irresponsible owners (e.g., aggressiveness towards both dogs and people, dog litter) can prevent both dog-owners and non-owners from using public spaces (sidewalks, parks).
Cutt H, Knuiman M, Giles-Corti B. Does getting a dog increase recreational walking? Int J Behav Nutr Phy. 2008;5(SS):1-10.	Quantitative: self-administered surveys at baseline and 12 months, completed by RESIDE participants who did not own a dog at baseline	Adults who did not have a dog at baseline– N = 773 (92 acquired a dog prior to follow-up, 681 did not) Suburban residents of new housing estates Perth, Western Australia	Physical urban environment - RESIDE Project (described above)	The group of participants that acquired a dog walked less at baseline, but more at follow-up, than the group that did not acquire a dog.<br><br>After acquiring a dog, new dog-owners increased neighborhood-based walking for recreation by 48 minutes/week on average.<br><br>New dog-owners increased total weekly walking (including walking for transport) by 38 minutes/week.<br><br>New dog-owners were more likely to report a perceived increase in neighborhood social cohesion than non-owners.	Increases in the amount of time spent walking each week point to the possibility that these new dog-owners (and their dogs) may contribute to the positive social environment of the community through being 'out and about', leading to social interactions and a general sense of community safety. Interestingly, new dog-owners also reported a perceived increase in neighborhood social cohesion after acquiring a pet dog.

Day R. Local environments and older people's health: Dimensions from a comparative qualitative study in Scotland. Health Place. 2008;14:299-312.	Qualitative: case studies involving semi-structured interviews and field observations; sample recruited from local community groups	Older adults (62 – 90 yrs) – N=45<br>Majority were 70+, living independently<br>Varied SES representation<br>Glasgow region of Scotland<br>(Authors did not disclose specific locations)	Physical urban environment – inner urban and suburban neighborhoods, and a small coastal town	Dog litter in public, suburban open spaces favored by dog-owners was identified as an environmental barrier to physical activity by older adults.<br><br>Dog litter posed the greatest issue in a suburban estate where parkland was situated in close proximity to houses.	Dog-owners who do not clean up after their dogs create an environmental nuisance for others who also wish to access local open spaces, contributing to negative perceptions and potentially inhibiting use of these areas for physical activity. Notably, the highest SES location had the most advantageous environment overall, while that in the lowest SES location had the most problematic environment.
Duncan, M., & Mummery, K. (2005). Psychosocial and environmental factors associated with physical activity among city dwellers in regional Queensland. Prev Med, 40(4), 363-372.	Quantitative: analysis compared associations using both self-report and objective, GIS-derived measures. Sample was randomly selected, and self-report measures were attained using a cross-sectional, computer-assisted telephone interview survey	Adults (18 – 94 yrs)<br>N=1,281<br>Residents of Rockhampton, Queensland, Australia	Physical and social urban environments – factors associated with attaining recommended levels of physical activity and/or participating in walking for recreation	Almost 60% of sample attained sufficient levels of activity (150 min/week).<br><br>Self-efficacy and social support were both positively associated with the likelihood of attaining sufficient levels of activity.<br><br>Number of active people within a 1 km radius was positively associated with attaining sufficient levels of activity.<br><br>Nearly 60% of the sample reported doing some recreational walking during the week prior to the survey. Females and respondents considered to be overweight were more likely to engage in walking than other respondents.<br><br>Social support was positively associated with walking for recreation; self-efficacy was not associated with walking for recreation.<br><br>People whose homes were in the middle tertile in terms of number of dogs within a 0.8 km radius were 66% more likely to walk than those whose homes were in the lowest tertile.	Living in a neighborhood where more people are being active seems to encourage increased activity as well for the entire sample – dog-walkers who are regularly active in their neighborhoods encourage non-dog-owners to be active as well.<br><br>Living in a neighborhood that has a larger canine population living within a 0.8 km radius (i.e., mid-compared to lowest-tertile) was correlated with a higher likelihood of walking among the sample, though dog-ownership may confound this finding (those sampled within such areas may be more likely to own and walk dogs). Regardless, this finding supports the potential for positive contributions of dogs in encouraging more neighborhood-based physical activity for both dog-owners and non-owners.
Evenson K, Sarmiento OL, Macon ML, Tawney KW, Ammerman AS. Environmental, policy, and cultural factors related to physical activity among Latina immigrants. Women Health. 2002;36(2):43-57.	Qualitative: 6 focus groups; sample recruited through a local health centre, apartment complex, and church	Adult women, first-generation Latina immigrants (20-50 yrs) – N=49<br>Not regular exercisers<br>Eastern and western counties in North Carolina, USA	Physical, social, and political urban environments – factors moderating physical activity patterns	Fear of unleashed dogs was highlighted as a safety concern which prevented some of the women in this study from walking outdoors in some neighborhoods.<br><br>Enforcement of leash laws was suggested by the participants as one form of policy-level intervention that, if implemented, might encourage increased participation in physical activity.	Fears of unleashed dogs prevented some of the study participants who did not own dogs from walking in their neighborhoods. The significance of this was underscored by the study participants' suggestion to address enforcement of leash laws as a means of encouraging more neighborhood-based activity.

Eyler AA, Vest JR, Sanderson B, Wilbur J, Matson-Koffman D, Evenson KR, et al. Environmental, policy, and cultural factors related to physical activity in a diverse sample of women: The Women's Cardiovascular Health Network Project-summary and discussion. Women Health. 2002;36(2):121.	Qualitative: 42 focus groups conducted in various locations across the USA	Adult women – N=305<br>Ethnic minority and low income populations – African American, American Indian, Latina, and White<br>Urban and rural representation from across the USA	Physical, social, and political urban environments – factors moderating physical activity patterns	Fear of being attacked by stray or loose dogs inhibited walking and other outdoor activities and indicated a low sense of personal safety within some neighborhoods. This was mentioned by both urban and rural participants.<br><br>Feeling disconnected from the community was highlighted as contributing to inactivity, particularly among Latina groups. Most non-white ethnic groups involved in this study highlighted the need for community support *via* shifted social norms.	Reported fears of stray or loose dogs were associated with lowered levels of physical activity and walking among non-dog-owners.<br><br>Fears of stray or loose dogs could contribute to feelings of being disconnected from the community, which were brought up by participants in some focus groups, and which are thought to exacerbate physical inactivity.<br><br>The barrier to being more physically activity that resulted from fears of unleashed dogs could also prevent the establishment of new, community-based norms of physical activity and walking.
Griffin SF, Wilson DK, Wilcox S, Buck J, Ainsworth BE. Physical activity influences in a disadvantaged African American community and the communities' proposed solutions. Health Promot Pract. 2008;9(2):180-190.	Qualitative: 3 focus groups, recruited through a local community center	Adult African-Americans – N=27<br>Majority were over 50<br>Majority were women<br>Residing in a small disadvantaged (low income, high crime) suburban community in southeastern South Carolina, USA<br>(Specific location was not disclosed by the authors.)	Physical and social urban environments – factors moderating physical activity patterns	Stray dogs were identified, primarily by women, as non-criminal factors that had a negative impact on the safety of the neighborhood, and that were barriers to being physically active.<br><br>Participants felt that increasing community connectedness and social support could be a primary factor in facilitating increased neighborhood-level physical activity, and further suggested that support and assistance between neighbors could build this.<br><br>Physical attributes such as lighting and sidewalks were also identified as amenities that would encourage more physical activity.	Fears of encountering stray dogs were identified as inhibiting neighborhood-based physical activity for non-dog-owners in this study.<br><br>At the same time, the social attributes of neighborhoods that were identified by participants as encouraging more physical activity could be enhanced by cultivating dog-ownership, and dog-walking, in the neighborhood. The physical attributes suggested to increase physical activity (lights, sidewalks) would also support dog-owners in walking their dogs.
Guéguen N, Ciccotti S. Domestic dogs as facilitators in social interaction: An evaluation of helping and courtship behaviors. Anthrozoos. 2008;21(4):339-349.	Quantitative: systematic field observations; random assignment of participants to experimental and control groups based on presence or absence of a dog	Adults (18 – 60 yrs) – N=440<br>Urban public settings (outdoor malls, bus shelters)<br>France<br>(Specific location was not disclosed by the authors.)	Social urban environment – presence of a dog as facilitating social interactions	A well-behaved, leashed dog accompanying a conventional-looking person was found to lubricate social interactions and resulted in a measurable increase in benevolent behavior when assistance was solicited in shared, public spaces.	The finding that dogs lubricated social interactions among strangers underscores the potential for dogs to contribute favourably to the social environment, in ways that could support physical activity for both dog-owners and non-owners.

Johnson, R. A., & Meadows, R. L. (2002). Older Latinos, pets, and health. West Nurs Res, 24(6), 609-620	Quantitative: cross-sectional, descriptive survey of Latinos, administered by bilingual surveyors in either English or Spanish language	Convenience sample of Latino dog-owners, 50 years and older, recruited from veterinary practices in a large southwestern American city<br>N=24<br>(Specific location was not disclosed by the authors.)	Social urban environment – health-related benefits of owning a dog	Two-thirds of this sample of older adult dog-owners reported that dogs kept them active<br><br>Half of the participants reported that they regularly walking or jogging with their dog.<br><br>Nearly half reported that they met new people because of their dog.	Respondents who owned dogs attributed their dogs with keeping them active.<br><br>Through meeting new people because of their dogs, participants might increase both quantity and quality of relationships with neighbors or others who the dog-owners encounter regularly when out with their dogs, contributing positively to the social environment of surrounding neighborhoods. This could support neighborhood-based physical activity for both the dog-owners and non-owners.
Johnson, R. A., & Meadows, R. L. (2010). Dog-walking: motivation for adherence to a walking program. Clin Nurs Res, 19(4), 387-402.	Mixed methods – quantitative: pre/post measures of BMI and walking status; qualitative: individual interviews	Adults (40 and older)<br>Recruited from 2 public housing facilities in a US Midwest city<br>N=26<br>Able to walk without human assistance, though canes and walkers were acceptable.<br>Socio-economically disadvantaged , primarily Caucasian with some African American participants<br>Participants were English-speaking, interested in exercise, and unafraid of dogs.	Social urban environment – adherence to an organized, supervised dog-walking program (dogs are provided, not owned)	The overall attrition rate from the study was 15%, which was markedly improved over other documented attrition rates for physical activity interventions (as high as 50%). Attrition was based on reasons other than walking.<br><br>At one site, the adherence rate to the walking program was 72% (this was for the 50-week program); at the other site, it was 53% (this was for the 26-week program). Only 4 of the original 30 participants withdrew without explanation: another 4 left the program for reasons including relocation, scheduling conflicts, and poor health.<br><br>Through interviews, it became apparent that adherence to the program was due to a sense of commitment they felt to the dogs. Regular comments suggested that participants perceived that the dogs were waiting for them, and depended upon them for exercise.<br><br>This dog-walking program was successful in sustaining increased levels of physical activity for this sample of older, socioeconomically disadvantaged adults. While participants did not own the dogs, nor did they walk with the same dog each day, they were motivated by the dogs' needs and thus able to maintain a reasonable walking regimen which enabled them to attain 100 minutes/week of moderate physical activity.	The sense of responsibility to care for a dog *via* walking is not necessarily contingent on ownership, and resulted in high levels of retained participation in an intervention designed to increase physical activity for a disadvantaged population.

King AC, Castro C, Wilcox S, Eyler AA, Sallis JF, Brownson RC. Personal and environmental factors associated with physical inactivity among different racial-ethnic groups of U.S. middle-aged and older-aged women. Health Psychol. 2000;19(4):354-364.	Quantitative: cross-sectional, telephone-administered survey of a stratified random sample of women in USA	Middle-aged and older women (40 yrs and older) from African American, Hispanic, and American Indian-Alaskan Native sub-groups, and White comparison group – N= 2,912<br>Urban and rural representation<br>USA	Physical and social urban environments – factors moderating physical activity patterns	African American and American Indian-Alaskan Native women were the most likely to report presence of unattended dogs in their neighborhoods and also had highest percentages of women categorized as sedentary re: physical activity level.<br><br>African American and American Indian-Alaskan Native women were the most likely to report presence of unattended dogs in their neighborhoods, and also were the most likely sub-populations to describe their neighborhoods as 'very unsafe'.<br><br>Awareness of unattended dogs was associated with more rather than less physical activity, which countered previous findings. The authors suggested that a heightened awareness of unattended dogs could reflect spending more time out in the neighborhood for this particular case. (Also see Chad et al.'s (2005) results).	Unattended dogs were positioned as contributing negatively to physical and social environments by adding to the perception that the neighborhood or community was not safe. This inhibited access to outdoor, neighborhood-based physical activity for non-dog-owners.
King AC, Toobert D, Ahn D, Resnicow K, Coday M, Riebe D, et al. Perceived environments as physical activity correlates and moderators of intervention in five studies. Am J Health Promot. 2006;21(1):24-35.	Quantitative: cross-sectional survey of available participants from randomized community samples participating in RCTs	Adults (18 – 65+) – N=769 from 5 different samples<br>Diverse representation (SES, education, ethnicity, health status)<br>Urban centres: Stanford, California; Eugene, Oregon; Atlanta, Georgia; Memphis, Tennessee; and Kingston, Rhode Island, USA	Physical and social urban environments – Behavior Change Consortium (BCC) Initiative	Reporting stray or loose dogs in one's neighborhood was negatively associated with minutes/week of moderate intensity (or more vigorous) physical activity (Georgia sample).<br><br>Reporting stray or loose dogs in one's neighborhood was negatively associated with minutes/week of walking for errands (Tennessee sample).<br><br>Reporting stray or loose dogs was negatively associated with minutes/week of walking for leisure (Georgia and Tennessee samples).<br><br>Presence of stray or loose dogs reportedly impeded attempts to become more physically active as part of a formal intervention for some experimental groups (Georgia and Tennessee).<br><br>Of note, Atlanta and Memphis samples were mid-range compared to the other samples regarding education and income, but were the only samples that were predominantly African American. These two experimental groups also had fewer single family homes in their neighborhoods.<br><br>Seeing or speaking with others was positively associated with minutes/week of physical activity.	Given negative associations between stray or loose dogs and walking for transport, presence of loose dogs in neighborhoods may have a negative impact on physical and social environments and discourage walking to some local amenities and services.<br><br>Seeing or speaking with others was positively associated with minutes/week of physical activity, but this social environmental factor may be absent in neighborhoods where stray/loose dogs discourage residents from being outdoors (i.e., the neighborhood is perceived as unsafe).<br><br>This is also a factor that could potentially be leveraged in conjunction with a campaign to increase responsible dog-ownership behaviors, including dog-walking.

Knight S, Edwards V. In the company of wolves: the physical, social, and psychological benefits of dog-ownership. J Aging Health. 2008;20(4):437-455.	Mixed methods: qualitative - 10 focus groups; quantitative - questionnaire was also used to describe socio-demographic variables of participants	Adult dog-walking owners (28 – 85 yrs, mean=60) – N=65<br>Recruited from 12 popular dog-walking sites<br>Hampshire countryside, south of England	Social urban environment – owning a dog as facilitating physical and social activity while using dog-walking facilities (parks and paths) and in suburban neighborhoods	Dogs were viewed by focus group participants as offering health benefits as a result of walking and exercising. To walk their dogs regularly, dog-owners in this sample overcame such barriers as individual mental and physical health issues, lethargy, bereavement, sense of insecurity or mistrust in neighborhood settings, and inclement weather. Participants reported inevitably feeling better for having gone out with their dog<br><br>Dogs also made participants feel safer, both at home and while out, and thus enabled them to override underlying personal safety concerns to go out walking. As a result, they were more likely to be regular neighborhood walkers and interact with others – dog-owners and non-owners – who were outdoors as well.<br><br>Participants noted that they felt more trust towards others out walking dogs than those out walking without dogs.<br><br>Participants attributed being out walking their dogs as a means of meeting other dog-walkers, making friends, and having a point of entry into a conversation. Dog-owners were motivated to walk by the likelihood of meeting and belonging to a network of other dog-owners with whom they had become acquainted through regular dog-walking. Interactions with other dog-owners/dogs were positive in nature.<br><br>There was a sense among participants that the dog was what sparked interactions with strangers and that without a dog, there would be no informal interactions.	While this sample of respondents was composed of dog-owners selected because they were also walkers, the results point to a dog's ability to over-ride certain barriers that might otherwise prevent the individual owner from walking regularly. These included physical illness and depression; inclement weather, and concerns about personal safety.<br><br>Findings also pointed to the importance of social interactions, both with other dog-owners and non-owners in the neighborhood, as part of the dog-walking experience.<br><br>These positive social encounters help create a physical and social environment that encourages neighborhood-based physical activity for dog-owners and non-owners alike. Such an environment contributes to social cohesion, which supports neighborhood-based physical activity.

Kowal J, Fortier MS. Physical activity behavior change in middle-aged and older women: the role of barriers and of environmental characteristics. J Behav Med. 2007;30:233-242.	Quantitative: surveyed at baseline and 6 months a sample recruited from community centres, community health centres, fitness clubs, and professional women's groups	Adult women (39-68 yrs) – N=149 Caucasian, married, educated, higher SES (50% > $75K/yr) Canadian urban centre (specific location was not identified by authors)	Physical and social urban environments – factors moderating physical activity patterns	Unattended dogs (and unsafe environments in general) were among the least reported barriers to being physically active by this sample, with more emphasis placed on amenities (sidewalks, street lights, enjoyable scenery, pathways and trails), social environments (seeing others walking or exercising), and topography (hills).<br><br>Those who reported increased or stable activity levels were more likely to report seeing others walking or exercising in their neighborhood.	This is an example of a study set in a population where fears of stray or loose dogs are minimal, likely due to the socio-cultural composition of the neighborhoods in which these participants are likely to reside (high income, Caucasian).<br><br>Participants who reported increased or stable activity levels were also more likely to report seeing others walking or exercising in their neighborhood. It is plausible that those 'others' could include regular dog-walkers, given the socio-demographics of the sample, although there is no direct evidence for this provided in the study.
Lee, H., Shepley, M., & Huang, C. (2009). Evaluation of off-leash dog parks in Texas and Florida: A study of use patterns, user satisfaction, and perception. Landscape and Urban Planning, 92(3-4), 314-324.	Quantitative: observations and behavior mapping were made at 4 locations; surveys were hand-distributed to park-users	Persons using 4 dog-parks located in Florida (1) or Texas (3) Questionnaires were completed by a sub-sample of adult dog-owners (25 – 54 years) N=267 Sample was predominantly Caucasion, married, highly educated, with annual household incomes between $60 and $120K	Physical and social urban environments: provision of neighborhood-based or regional off-leash dog parks	Dog parks located close to residential neighborhoods were used most frequently and were more likely to be accessed on foot as opposed to by car.<br><br>In a new community with a dog park, nearly 95% of the participants walked to the park. About a third visited the park daily, and a quarter reported socializing with neighbors while using the park. Over 75% of the sample agreed that dog parks provided opportunities to meet neighbors and build a sense of community through socializing with others.<br><br>In a dog park with a water feature, nearly 25% of users were observed participating in mobile activities, significantly more than in other dog parks without water features. The linear shape of this park also encouraged exercise-walking and interactions with other users.<br><br>Some participants identified irresponsible owners and other dogs' behaviors as constraints to visiting dog parks.<br><br>After dog parks, the most frequented locations for dog-related outdoor activity were: walking around the neighborhood with the dog on-leash, leaving the dog off-leash in an enclosed backyard, and walking the dog on-leash to a nearby park.	Dog parks as nearby destinations initiated neighborhood-based walking for dog-owners, who were more likely to walk their dogs to and from the dog park. This results in a regular, visible presence of dog-owners out walking, which can encourage others (dog-owners and non-owners) to walk more as well.<br><br>The finding that walking in neighborhoods and local parks were frequent activities of dog-walkers suggests that dogs are a regular presence in those settings, with the potential to impact the social environment in either positive or negative directions, depending upon whether responsible dog-ownership is being practiced.<br><br>In the dog parks in this study, the onus was upon owners to pick up after their dogs and keep them under control. Self-policing rather than formal by-law enforcement predominated, which might place at a disadvantage persons who are not comfortable reprimanding others in public. At the same time, it might raise awareness and practice of responsible dog-

					ownership behaviors through informal exchanges and observation of others.
Lloyd K, Burden J, Kiewa J. Young girls and urban parks: Planning for transition through adolescence. Journal of Park and Recreation Administration. 2008;26(3):21-38.	Qualitative: semi-structured interviews with a sub-sample of previous focus group participants referred by local teachers and youth agencies	Adolescent girls (13 – 18 yrs) – N=11<br>Middle-class, family-oriented suburb "The Gap", Brisbane, Australia	Physical urban environment – neighborhood parks	Access to parks as inviting destinations provided the study participants with opportunities for both escape and socialization – dogs (walking, or playing) were mentioned regularly in the context of park use.	Dogs facilitated walking for some of the girls who participated in this study. Having youth in a local park could plausibly have either positive or negative impact on the social environment, depending upon activities and behaviors being witnessed; e.g., a girl walking or playing fetch with a well-behaved dog might be viewed more favourably than a group of teens taking over playground equipment when families with younger children are trying to use these amenities.
Martinez S, Arredondo E, Perez G, Baquero B. Individual, social, and environmental barriers to and facilitators of physical activity among Latinas living in San Diego County - focus group results. Fam Community Health. 2009;32(1):22-33.	Qualitative: 2 focus groups with a convenience sample selected from two Catholic churches	Latina women immigrants (18 – 65 yrs) – N=25<br>Lower household income (50% <$1,500/month)<br>Lower education levels (84% less than high school education)<br>Residing in border communities in San Diego County, USA	Physical and social urban environments – factors moderating physical activity patterns	Being chased by neighbors' unleashed dogs prevented some of the study participants from walking in their neighborhood.<br><br>Participants also brought up issues with the perceived safety of local public spaces, for example those used by drug dealers, or settings where a rape had taken place.	These findings highlight the connection between the characteristics of the physical and social environments in which the participants reside and the likelihood of dogs (unleashed or stray) acting as barriers rather than facilitators of physical activity for some non-owners. There appears to be a connection between perceiving that the neighborhood is unsafe and fears of encounters with uncontrolled (loose or stray) dogs.

McNicholas J, Collis GM. Dogs as catalysts for social interactions: Robustness of the effect. Br J Psychol. 2000;91(1):61.	Quantitative: systematic field observation – assignment of participants to experimental and control groups based on presence/absence of a dog	Adults using a variety of public spaces (dropping off children at school, public transit, university campus, city centres)<br>N=206 encounters (Exp 1);<br>N=1,170 encounters (Exp 2)<br>Conducted in a small English city in the UK (the identity was not disclosed by the authors)	Social urban environment – presence of a dog as facilitating social interactions	The effect of a dog as a catalyst for social interactions was found to be robust, and was not confined to areas commonly associated with dog activities, such as parks and walking destinations.<br><br>Interactions were initiated by the strangers, not the dog-handler or the dog, and may plausibly contribute to a social environment that supports walking and other forms of local activity through sense of community and social cohesion.<br><br>This effect was particularly marked for interactions between strangers, thus is not limited to those with established relationships. However, acquaintances (persons slightly known to the dog-handler) whose initial exchanges were prompted by the dog were observed to continue to converse with the handler, even after the experiment was over and the dog was no longer present, suggesting that the effect of the dog had long-term influence on the quality of these social relationship.	This study offers further evidence that the presence of a dog somehow invites others (e.g., non-owners) to engage with a person who is accompanied by a non-threatening dog. This does not depend on familiarity, or even being in a setting where dogs are expected.<br><br>Informal social encounters between strangers can contribute positively to the perceived social environment, ultimately supporting increased physical activity levels for dog-owners and non-owners alike.<br><br>This study also provides evidence that having a dog as a point of entry can increase the quality of a weak, existent relationship, as might exist between neighbors. Strong neighbor relationships are thought to contribute to social cohesion, supporting neighborhood-based physical activity.
Peel, E., Douglas, M., Parry, O., & Lawton, J. (2010). Type 2 diabetes and dog walking: patients' longitudinal perspectives about implementing and sustaining physical activity. The British Journal of General Practice: The Journal of the Royal College of General Practitioners, 60(577), 570-577.	Qualitative: repeat interviews at baseline, 6 months, 1 year, and 4 years to explore perceptions, experiences, and adjustments made to diabetic condition during post-diagnostic period	Adult patients recruited within 6 months of receiving a clinical diagnosis of type 2 diabetes and who gave permission for re-contact following 1st year of study<br>Purposive selection to represent demographics of type 2 diabetics in Lothian, Scotland.<br>N=20	Social urban environment – dogs as motivators of sustained physical activity	For the few participants who had successfully implemented and maintained increases in physical activity levels, walking dogs played a vital role. Dogs provided an extrinsic incentive to walk, allowing some of the patients to overcome an intrinsic lack of motivation or dislike of physical activity. Furthermore, for some, the dog was attributed with facilitating increased gains in physical activity compared to participating without a dog.<br><br>One participant reported that the company of a recently-acquired dog augmented existing physical activity enjoyment and participation.<br><br>For one participant who did not own a dog, the social aspect of regularly joining a neighbor's daily dog-walk was both preferable and more effective than any other attempts she had made to become more physically active.	The ability of a dog to motivate regular, maintained walking was not limited to dog-owners alone. A non- owner was aided by a dog – through joining a neighbor's dog-walking regime – in finding an enjoyable and regular means of increasing physical activity levels through walking.<br><br>The lack of success in finding and maintaining a regular physical activity program reported by study participants who did not own or have access to a dog is striking.

Sallis J, King A, Sirard J, Albright C. Perceived environmental predictors of physical activity over 6 months in adults: Activity Counseling Trial. Health Psychol. 2007;26(6):701-709.	Quantitative: prospective cohort study of participants in Activity Counseling Trial (ACT), recruited from primary care facilities	Adults (35 – 75 yrs) – N=861<br>Demographically diverse - urban<br>Stable health<br>Participants in ACT intervention<br>Stanford, California; Memphis, Tennessee; and Dallas, Texas, USA	Physical and social urban environments – Activity Counseling Trial (ACT), a physical activity intervention targeting physically inactive adults with no history of coronary heart disease	Women who reported loose or unattended dogs in their neighborhoods also reported approximately 50 minutes/week less physical activity than women who did not report this barrier.<br><br>Fear of bodily harm (dog attacks, other criminal activities) reduced physical activity levels for women.<br><br>Fear of loose or stray dogs was indicative of the perception of an unsafe neighborhood.	Women were more likely to report seeing unattended dogs, and were less likely to report feeling safe walking in their neighborhoods, compared to men.<br><br>There was a significant decrease in the activity levels of women who reported unattended dogs in their neighborhoods, thus this group of non-owners was particularly sensitive to the negative effect dogs can have on social environments. The authors noted that this association was not explained by variations in SES.
Sanderson B, Littleton M, Pulley L. Environmental, policy, and cultural factors related to physical activity among rural, African American women. Women Health. 2002;36(2):75-90.	Qualitative: 6 focus groups recruited by a community-based project coordinator via newspaper ads, flyers, and word-of-mouth	African American women (20 – 50 yrs) – N=61<br>Majority (66%) considered obese; 40% below poverty level;<br>Not regular exercisers<br>Residing in a rural community in Wilcox County, SW Alabama, USA	Physical and social rural environments –factors moderating physical activity patterns	Low levels of neighborhood-based walking were attributed to personal safety concerns, including unleashed or stray dogs that 'bothered people'.<br><br>Seeing no one else in the neighborhood being active was identified as a barrier to becoming more active.<br><br>Poor relationships with neighbors were also thought to contribute to physical inactivity within the neighborhood.<br><br>Positive feelings about neighbors or neighborhoods seemed to increase feelings of safety while exercising outdoors<br><br>Enforcement of animal control policies was suggested by participants as an important intervention to increase levels of physical activity.	Having 'a good neighborhood' and a situation where people look out for one another facilitated exercising outdoors *via* feelings of safety: these factors have been linked positively with dog-walkers in some studies.<br><br>The need to address the negative fears of loose dogs was raised in this study, in order to leverage responsible dog-owner behaviors in ways that can benefit the physical and social environments, leading to increased physical activity for all.<br><br>Seeing no one else out being active was also a barrier; if there were dog walkers in the neighborhood, then there would be a visible level of activity

Thorpe, J., Kreisle, R. A., Glickman, L. T., Simonsick, E. M., Newman, A. B., & Kritchevsky, S. (2006). Physical activity and pet ownership in Year 3 of the Health ABC Study. J Aging Phys Ac, 14(2), 154-168.	Quantitative longitudinal study: face-to-face administered questionnaire at year 4 of the Health ABC Study	Older adults, aged 70 – 79 (participating in Health ABC Study) Randomly sampled from White Medicare beneficiaries and Black community-dwelling residents from Memphis, TN or Pittsburgh, PA reporting no difficulty walking 1/4 mile or climbing 10 stairs without resting. Exclusion criteria included problems with activities of daily living, cognitive impairment, difficulty communicating with interviewer, intention of moving within 3 years, and active cancer treatment over past 3 years. (N=2,533; $N_{dog-owners}$=396)	Social urban environment – owning a dog as a motivator of sustained physical activity	Dog-owners reported higher levels of non-exercise-related walking (defined as walking to the store, to church, walking the dog) compared to non-pet owners and pet owners who did not own dogs. Dog-owners also reported greater frequency and duration of walks than other sub-groups.	As dog-owning older adults participated in more frequent neighborhood-based walks, and of longer duration, they are likely to be a visible, regular presence out in their neighborhood. This could encourage others walk, and could contribute positively to the social environment in ways that could encourage increased walking among non-dog-owners and owners alike.
Thorpe, R. J., Simonsick, E. M., Brach, J. S., Ayonayon, H., Satterfield, S., Harris, T. B., Garcia, M., et al. (2006). Dog-ownership, walking behavior, and maintained mobility in late life. J Am Geriatr Soc, 54(9), 1419-1424.	Quantitative longitudinal study: face-to-face administered questionnaire and objective measures of mobility at years 3 and 6 of the Health ABC Study	Older adults, aged 70 – 79 (participating in Health ABC Study, as described above) N=2,533; $N_{dog-owners}$=394	Social urban environment – owning a dog as a motivator of sustained physical activity	At baseline, dog-walkers' walking behaviors were similar to those of non-dog-owners who walked equivalent amounts. After adjusting for demographics, both of these groups were seven times more likely than non-walking dog-owners to achieve 150 minutes of walking per week.<br><br>Three years later, those participants who were dog-walkers at baseline were twice as likely as any others (including the walking non-dog-owners at baseline) to meet the recommended levels of walking.	All "non-exercise-related walking" examples took place in the neighborhood, with the possible exception of dog-walking, which was unspecified in location. Thus, older adults who owned dogs were more likely to be out consistently (i.e., over several years and possibly using similar routes) compared to non-dog owning older adults. This could potentially have a positive impact on the social environment in terms of getting to know neighbors, having social interactions, and keeping an informal eye on the neighborhood.<br><br>The direction of influence on the social environment is dependent upon whether dog-owners are compliant with responsible behaviors of keeping dogs leashed and picking up dog litter.

Webley P, Siviter C. Why do some owners allow their dogs to foul the pavement? The social psychology of a minor rule infraction. J Appl Soc Psychol. 2000;30(7):1371-1380.	Quantitative: systematic field observations as well as a quantitative intercept interview and self-administered questionnaire	Dog-owners – $N_{observed\ incident}$=101; $N_{questionnaire}$=87<br>Sampled dog-walkers observed in 3 public parks and 2 public, paved sidewalk locations<br>Signage about cleaning up after dogs present in all locations<br>Disposal bins for dog litter present in all locations but one<br>Exeter and Highcliffe, England	Physical urban environment – parks and paved public sidewalks with clear signage regarding dog litter as well as disposal facilities	An awareness of social norms surrounding responsible dog-ownership as well as physical location were both found to be somewhat predictive of responsible *vs.* irresponsible behavior of owners in terms of cleaning up after their dogs.<br><br>The majority of the observed dog-walkers in this study cleaned up their dog's litter: 70% of those observed walking their dogs in parks cleaned up the dog's litter. However, only 53% cleaned up after their dogs when walking on local sidewalks.	Compliance with cleaning dog litter on paved sidewalks was considerably lower than in parks, suggesting that some might avoid using neighborhood sidewalks, an important built-environment factor associated with neighborhood-based walking behavior, due to the nuisance caused by the presence of dog litter.
Wells DL. The facilitation of social interactions by domestic dogs. Anthrozoos. 2004;17(4):340-352.	Quantitative: systematic field observation – assignment of participants to experimental and control groups based on presence/absence of stuffed animal, houseplant, or different breeds of dogs	Adult pedestrians – N=1,800 (300 x 6 experimental conditions)<br>Belfast, Northern Ireland, UK	Social urban environment – presence of a dog as facilitating social interactions	Presence of a dog facilitated more social interactions between strangers (eye contact, a smile, or a conversation) compared to other experimental or control conditions. Persons walking alone were more likely to interact with the experimenter than those in pairs or groups.<br><br>All individuals who initiated conversations also stroked the dog or puppy.<br><br>Females who were walking alone were the most likely to interact in some way (verbal or non-verbal) with the researcher who was walking either a puppy or an adult Labrador. The puppy garnered the longest conversations.<br><br>Walking accompanied by a Rottweiler (perceived as an aggressive breed) garnered the fewest acknowledgements, of the three different dog breed/age scenarios.	Public spaces where popular breeds of dogs are commonly seen may have the most potential for maximizing the social interactions facilitated by dog-walking. This may contribute to a social environment that supports neighborhood-based physical activity.<br><br>This also underscores the importance for dog-owners with breeds perceived as aggressive in nature to comply with responsible dog-ownership practices in order to gain the trust of others, both dog-owners and non-owners.
Wells DL. Factors' influencing owners' reactions to their dogs' fouling. Environ Behav. 2006;38(5):707-714.	Quantitative: systematic field observations on owner's response to dog fouling in 8 public parks; questionnaire used to collect demographic data from owners	Lone walkers of a single dog – N=400 (8 sites x 50 dog walkers)<br>Public parks with anti-dog-fouling signage<br>Winter days (dry weather)<br>Belfast, Northern Ireland, UK	Physical and social urban environments – areas designated for off-leash use in parks that were outfitted with 'poop and scoop' bylaw signs, bags, receptacle containers, and dog toilets	Only half of all dog-owners in this study (53%) were observed to clean up after their dogs.<br><br>Men were less likely than women to clean up after their dogs, but age did not predict clean-up behaviors.<br><br>Dog-owners reporting lower socioeconomic status (SES) were less likely to clean up after their dogs than those in higher socioeconomic brackets.<br><br>Dog-owners with their dogs kept on-leash were more likely than those with dogs running loose to pick up after their dogs.	This study offers an example of how a social, contextual factor (e.g., SES) can help predict the direction of the contribution dogs can make to physical and social environments, which in turn are associated with physical activity.<br><br>Specifically, dog litter is less likely to be an environmental barrier to physical activity in higher SES neighborhoods, where it is more likely to be picked up, compared to lower SES neighborhoods, where it is more likely to be left behind. Dog-owners who kept their dogs

					on-leash were more likely to pick up after their dogs compared to those whose dogs were off-leash. Dogs that are off-leash may be perceived as uncontrolled as well, and thus the combined impact of both dog litter and uncontrolled dogs might work together to discourage physical activity for non-dog-owners, and other dog-owners, in areas where dogs are known to be off-leash.
Westgarth C, Pinchbeck GL, Bradshaw JWS, Dawson S, Gaskell RM, Christley RM. Dog-human and dog-dog interactions of 260 dog-owning households in a community in Cheshire. The Veterinary Record. 2008;162(14):436-442.	Quantitative: doorstep survey used to recruit dog-owners to participate in a cross-sectional mail-in survey	Adult dog-owners – N=279<br>Suburban – medium/low density housing with access to public facilities (parks, sports fields, wildlife reserve, agricultural land)<br>Cheshire, UK	Social urban environment – presence of a dog as facilitating social interactions with other humans and dogs	The majority of dogs in this study were walked at least once per day, and were walked both on- and off-leash.<br><br>Owners estimated that dogs met and interacted with 3-5 other people per day, outside of the household. Over 3/4 of dogs often or sometimes interacted physically with people when outside the household. Among household members, the types of dog-person interactions reported included nudging with the nose, licking hands, and jumping up, however the nature of interactions with people outside the household were not described.<br><br>Over 3/4 of dogs also interacted with other dogs, with interactions ranging from being playful to ignoring to being aggressive.<br><br>A substantial majority of dogs were confined to a secure area and never allowed to roam, and a small proportion of dogs were never allowed off-leash. When dogs were allowed off-leash, the majority were reported to remain in sight of their owner. As well, most owners reported always picking up their dog's litter when out walking.<br><br>Nearly 2/3 of dogs were walked at least once per day, and the duration of walks was reported to range from 16 minutes to 1 hour in length. Approximately half of dog-owners walked their dogs at regular times each day and in the same places each day. Over one-quarter of dog-owners never took their dogs out of their local area to walk. Furthermore, nearly all dog-owners reported seeing the same people (and their dogs) at least sometimes when walking their own dog.	Most of the dog-owners in this sample were regular dog walkers.<br><br>The frequency and regularity of daily dog walks suggest that social interactions with neighbors may result, as could the informal role of keeping an eye on the neighborhood, leading to improved social cohesion and supporting increased neighborhood-based physical activity for non-owners and other dog-owners.<br><br>This study highlighted the possibility of both friendly and aggressive physical interactions between dogs and people who are not their owners, as well as between dogs belonging to others. When these interactions are not positive, the potential exists to inhibit physical activity for the recipient of the dogs' aggressive behavior.

Wood L, Giles-Corti B, Bulsara M. The pet connection: pets as a conduit for social capital? Soc Sci Med. 2005;61(6):1159-1173.	Quantitative: cross-sectional telephone survey of a randomly selected sample	Adults (18+ yrs) – N=339<br>Three suburbs of comparable SES (residents for minimum 1 year)<br>Perth, Australia	Social urban environment - dogs and dog-owners in neighborhoods	The majority of dog-owners reported that owning a dog encouraged them to go for more walks in their suburb than they would otherwise do, and many dog-owners identified their dogs as a source of motivation to use community facilities such as parks and open spaces.<br><br>More dog-owners reported giving and receiving neighborly favours compared to non-pet owners, including favours that did not directly involve pet care.<br><br>Dog-owners also identified more people in their neighborhood to whom they could turn in a crisis or for support. Furthermore, dog-owners were less likely to report feeling lonely and more likely to report ease in getting to know people.<br><br>Half of dog-owners got to know people in their suburb via their dogs. Over 80% of dog-owners spoke with others while they were out walking their dogs. Dog-owners were more likely to perceive that neighbors would greet each other while out walking or in their yards/gardens.<br><br>Dog-owners attributed feelings of personal safety to their dog, both when out walking and when in their homes.<br><br>Dog-owners tended to score higher than non-owners on measures of social capital and sense of community.	Dogs were attributed with increasing neighborhood-based physical activity for their owners.<br><br>Dog-owners also attributed their dogs with helping them get to know people in their neighborhood. These social relationships benefit non-dog-owners and dog-owners alike, and create an environment that is more conducive to neighborhood-based physical activity for all residents.<br><br>For non-dog-owners, the familiarity resulting from repeat encounters with regular dog-walkers could increase sense of cohesion in general, benefitting both owners and non-owners in terms of supporting neighborhood-based physical activity through a positive social environment.

Wood LJ, Giles-Corti B, Bulsara MK, Bosch DA. More than a furry companion: The ripple effect of companion animals on neighborhood interactions and sense of community. Society & Animals. 2007;15(1):43-56.	Mixed methods: qualitative –12 focus groups conducted at community centres; quantitative results previously reported in Wood et al. 2005	Adults (18+ yrs) – N=86<br>Male and female participants recruited to represent young singles; workers with dependent children; stay-at-home parents with dependent children; retired persons<br>Three suburbs of comparable SES (residents for minimum 1 year)<br>Perth, Australia	Social urban environment - dogs and dog-owners in neighborhoods	Owning a dog increased the likelihood of dog-owners meeting other people (dog-owners and non-owners) in their community, both on their street and further out within their neighborhood. The presence of a dog was perceived as 'break[ing] the ice' and encouraging social interactions.<br><br>Participants without dogs also commented on the social opportunities that resulted from having dog-walkers out – notably, one such comment came from a retired person.<br><br>Dogs were portrayed by several participants as 'companions on duty,' accompanying their owners as they kept an eye on the neighborhood. Several participants considered seeing people 'out and about' in their neighborhoods, including dog-walkers, to be a positive indicator of community safety.	Both dog-owners and non-owners who participated in this study identified dogs as contributing positively to the social environment of their neighborhood as a result of being walked regularly, which led to getting to know one-another.<br><br>Regular dog-walking was also attributed with improving the perceived safety in the neighborhood, given that owners were out regularly and able to keep an eye on the neighborhood. This has also been identified as a correlate of physical activity in neighborhoods.<br><br>Thus dogs were positioned as contributing to a neighborhood environment that was conducive to increased neighborhood-based physical activity for all.
Ziersch AM, Baum FE, Macdougall C, Putland C. Neighborhood life and social capital: the implications for health. Soc Sci Med. 2005;60(1):71-86.	Mixed methods: quantitative cross-sectional postal survey to a random sample of adults; qualitative semi-structured interviews with willing individuals who had completed postal survey (data collected as part of a broader study, the Health Development and Social Capital Project (HDSCP))	Adults (18+ yrs) – N_{quant}=2400; N_{qual}=40<br>Below-average SES, but with pockets of advantage and disadvantage<br>Population is older-than-average and has a higher-than-average concentration of migrants and those for whom English is a 2nd language<br>Located in the Western region of Adelaide, Australia	Physical and social urban environments – built environment and composition of neighborhoods	Pets facilitated reciprocal favours between neighbors (taking care of each other's pets).<br><br>Local green spaces contributed to physical activity by providing destinations where people could gather and engage in activities including playing ball, walking dogs, using playground equipment, and others. As such, shared public green space was positioned as a place that attracts dog-owners and others, offering opportunities to socialize on neutral territory.	In this study, dogs were mentioned by participants as a part of the equation that ultimately translates into a more cohesive community (a 'healthy' community), and which can support neighborhood-based physical activity for both dog-owners and non-owners.<br><br>The presence of dogs was mentioned by non-dog-owners as a desirable component of a multi-use green space.
